# Cognitive Impairment and Brain Imaging Characteristics of Patients with Congenital Cataracts, Facial Dysmorphism, Neuropathy Syndrome

**DOI:** 10.1155/2015/639539

**Published:** 2015-04-28

**Authors:** Teodora Chamova, Dora Zlatareva, Margarita Raycheva, Stoyan Bichev, Luba Kalaydjieva, Ivailo Tournev

**Affiliations:** ^1^Clinic of Neurology, University Hospital “Alexandrovska”, 1431 Sofia, Bulgaria; ^2^Department of Diagnostic Imaging, University Hospital “Alexandrovska”, Medical University, 1431 Sofia, Bulgaria; ^3^National Genetics Laboratory, Molecular Medicine Center, Medical University, 1431 Sofia, Bulgaria; ^4^Harry Perkins Institute of Medical Research and Centre for Medical Research, The University of Western Australia, Nedlands, Perth, WA 6009, Australia; ^5^Department of Cognitive Science and Psychology, New Bulgarian University, 1618 Sofia, Bulgaria

## Abstract

Congenital cataracts, facial dysmorphism, neuropathy (CCFDN) syndrome is a complex autosomal recessive multisystem disorder. The aim of the current study is to evaluate the degree of cognitive impairment in a cohort of 22 CCFDN patients and its correlation with patients' age, motor disability, ataxia, and neuroimaging changes. Twenty-two patients with genetically confirmed diagnosis of CCFDN underwent a detailed neurological examination. Verbal and nonverbal intelligence, memory, executive functions, and verbal fluency wеre assessed in all the patients aged 4 to 47 years. Brain magnetic resonance imaging was performed in 20 affected patients. Eighteen affected were classified as having mild intellectual deficit, whereas 4 had borderline intelligence. In all psychometric tests, evaluating different cognitive domains, CCFDN patients had statistically significant lower scores when compared to the healthy control group. All cognitive domains seemed equally affected. The main abnormalities on brain MRI found in 19/20 patients included diffuse cerebral atrophy, enlargement of the lateral ventricles, and focal lesions in the subcortical white matter, different in number and size, consistent with demyelination more pronounced in the older CCFDN patients. The correlation analysis of the structural brain changes and the cognitive impairment found a statistically significant correlation only between the impairment of short-term verbal memory and the MRI changes.

## 1. Introduction

Congenital cataracts, facial dysmorphism, neuropathy (CCFDN) syndrome (OMIM 604168) is a complex autosomal recessive multisystem disorder, observed in patients of Gypsy ancestry [1–6]. All the affected are homozygous for the same IVS6+389C>T mutation in the* CTDP1* gene (Carboxy-Terminal Domain Phosphatase 1), mapping to 18qter and encoding the protein phosphatase FCP1 (transcription factor IIF-associating CTD phosphatase 1) [3, 7].

The clinical manifestations of the disease include congenital cataracts, facial dysmorphism, primary hypomyelination of peripheral nerves, growth delay, intellectual impairment, and hypogonadism [1, 5]. Early-onset congenital cataracts associated with microcornea, microphthalmos, and micropupil are essential ocular features of the CCFDN syndrome observed in the first days of life [8]. Facial dysmorphism, characterised by a prominent midface, thickening of the perioral tissues, forwardly directed anterior dentition, and micrognatia, becomes more evident during childhood. A progressive hypo/demyelinating predominantly motor peripheral neuropathy develops during childhood and adolescence [1, 6]. Other neurological abnormalities such as bilaterally extensor plantar responses, choreiform movements, cerebellar involvement of variable severity with ataxia, nystagmus, intention tremor, and dysmetria have been described in some of the cases [1, 8]. Recurrent parainfectious rhabdomyolysis may be an integral part of the CCFDN phenotype, reported in an increasing number of patients [2, 4–6].

Mild cognitive deficit was found to be invariably present in CCFDN patients. The cognitive impairment was thought to be nonprogressive [1, 9].

Cerebral and spinal cord atrophy, ventricular enlargement, and other less common white matter (WM) abnormalities have been described on brain MRI [1, 5, 9]. The changes were thought to be age related. A study using diffusion tensor MRI in a small group of CCFDN patients revealed a higher apparent diffusion coefficient and lower fractional anisotropy in the vermis and medulla oblongata, suggesting axonal loss in these parts of the brain [10].

The aim of the current study is to evaluate the degree of cognitive impairment in a cohort of 22 patients with CCFDN as well as its correlation with patients' age, motor disability, ataxia, and brain MRI changes. The study complies with the ethical guidelines of the institutions involved.

## 2. Materials and Methods

Twenty-two patients, 11 males and 11 females, homozygous for the CCFDN mutation in* CTDP1*, were admitted to the Clinic of Neurology at the University Hospital Alexandrovska, Sofia, for detailed physical and neurological examinations.

### 2.1. Clinical Evaluation

All the patients underwent thorough neurological examination. Motor impairment was evaluated by the Functional Disability Scale (FDS) [12]. The Klockgether ataxia score was used for measuring dysarthria, ataxia of walk, stance, upper and lower extremities, dysdiadochokinesis, and intention tremor on a scale from 0 to 5 (0: best, 5: worst) [13].

### 2.2. Neuropsychological Tests

All affected subjects underwent a formal neuropsychological assessment, including the following tests: general intelligence assessment by Wechsler Intelligence scale (HAWIK-R, Bulgarian version, based on WISC-R); memory tests (Rey Auditory Verbal Learning Test, including subtests of immediate and delayed recall and forced-choice recognition) [14]; language tests (Phonological Word Fluency and Semantic Word Fluency); and frontal and executive tasks (Tower of London), [11]. All tests were scored according to standard procedures as outlined in test manuals. Bearing in mind that all affected individuals belonged to an isolated ethnic group of loweducational and economic status, the results were compared to the results of a control group of 24 age and sexmatched healthy subjects with the same social and educational background.

### 2.3. Neuroimaging

Brain magnetic resonance imaging (MRI) was performed in 20 patients, using a 1.5 TMR imager (MR Signa HDxt, GE Healthcare, Milwaukee, USA). The examination protocol consisted of three series: sagittal spin-echo T1-weighted images (440–620/9–14 [TR/TE]), axial turbo spin-echo T2-weighted images (3520–5000/106–115 6 [TR/TE]), and coronal FLAIR images (8002–11002/120–140 [TR/TE]). The average section thickness was 5 mm. In 5 patients, coronal 3D SPGR T1-weighted and coronal T2-weighted images were obtained. Although all images were evaluated, scoring was based on axial T2-weighted and coronal FLAIR images.

The MRI changes of the CCFDN patients were evaluated by a scoring system, previously used for metachromatic leukodystrophy [15], adapted for CCFDN patients, with a maximal result of 33 points, corresponding to the most severe changes (Table 1). This approach allowed the evaluation of WM involvement, as well as the presence of cerebral and cerebellar atrophy. The hyperintense WM lesions were graded as 0 (absent), 1 (faint), or 2 (dense). The severity of global brain atrophy was measured as follows: 1 point: ventricular enlargement or widening of outer cerebrospinal fluid (CSF) spaces, 2 points: inner and outer cerebrospinal fluid (CSF) space widening, and 3 points: severe ventricular enlargement. The 3rd point was added in order to discriminate between moderate and severe ventricular enlargement.

### 2.4. Statistical Analysis

Statistical analyses were conducted using statistical package IBM SPSS Statistics 19.0. A *p* value below 0.05 was considered significant. The following statistical methods were used: descriptive statistics; Student's *t*-test, for testing hypothesis for difference between two independent and correlation analyses.

## 3. Results

The mean age of assessment of the CCFDN patients was 23.5 ± 13.66 years, ranging between 4 and 47 years (Table 2).

Although pregnancy and delivery were reported as uneventful, developmental delay was observed in all cases. The mean age of starting to walk was 2.76 ± 1.29 years, with only two patients reported to have started walking within normal limits and one who never acquired independent ambulation. The mean age of starting to talk was 2.98 ± 2.15 years with broad variation between 1.3 and 12 years (Table 2).

In all psychometric tests, evaluating different cognitive domains, CCFDN patients had statistically significant lower scores when compared to the healthy control group (Table 3).

Eighteen affected were classified as having mild intellectual deficit, whereas 4 had borderline intelligence. Language abilities were developed at a concrete and pragmatic level. Different cognitive domains (verbal memory, executive functions, and language skills) seemed similarly affected. Patients were socially integrated, but for their everyday functioning they needed guidance and assistance. No significant correlation was found between the cognitive impairment and the age of assessment (Table 4). There was no clear-cut correlation between the results of the cognitive tests and the severity of motor involvement, measured by FDS (Table 4).

In five patients, we observed other symptoms of central nervous system involvement in addition to the cognitive impairment, such as pyramidal signs in two, choreiform hyperkinesia in one, and mild cerebellar ataxia with Klockgether ataxia score between 7 and 11/35 in five.

Brain MRI demonstrated abnormalities in 19 out of 20 investigated patients (95%). The MRI findings are summarized in Table 5. All adult patients (11) in our group have gained complete myelination.

**Figure 1:**
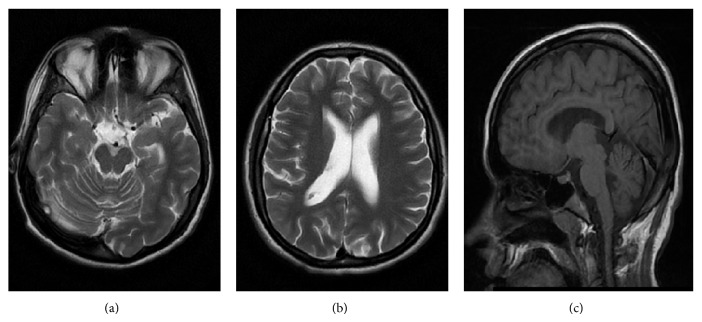
Patient number 12: a 26-year-old woman with cerebral and cerebellar atrophy and severity score 6. (a), (b) Axial T2W images show global cerebral atrophy with dilatation of subarachnoid spaces and ventricular system. Dilatation of interfoliar cistern as a consequence of cerebellar atrophy. (c) Sagittal T1W image demonstrates widening of subarachnoid spaces supratentorial and infratentorial.

Cerebral atrophy with enlargement of the lateral ventricles and dilatation of the subarachnoid spaces were observed in 18 of the affected (90%). Two affected patients (patients number 1 and number 3), belonging to the age group 4–8 years, had no signs of cerebral atrophy. Ventricular enlargement and cortical atrophy were evaluated as mild in seven patients, as moderate in six, and as severe in five. Considering the brain atrophic changes most of the patients had diffuse brain atrophy with dilatation of subarachnoid spaces and ventricular system (14/20) (Figure 1). In those without ventricular enlargement parietal and frontal subarachnoid spaces particularly close to the convexity were dilated with exception of patient number 6 with dilated subarachnoid spaces in frontal, temporal, and parietal region. Cerebellar atrophy was found in only one patient, who also had global cerebral atrophy with inner and outer CSF space widening. Four patients had white matter bulk loss. A periventricular rim was observed in 13 and thin corpus callosum in three.

**Figure 2:**
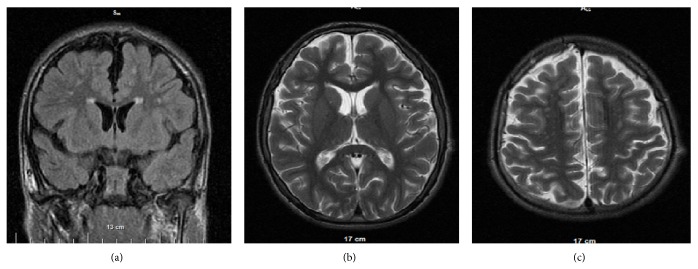
Patient number 7: a 13-year-old boy with severity score 7. (a) Coronal FLAIR image demonstrates dilated subarachnoid spaces, periventricular dense rim, and dense subcortical and periventricular foci in frontal lobes. (b), (c) Axial T2W image shows bilateral frontal subcortical hyperintensities (dense: 2 points), dilatation of subarachnoid spaces, and no significant dilatation of ventricular system (severity of global brain atrophy: 1 point).

Hyperintense lesions, varying from small single to multiple diffuse, encompassing periventricular white matter and brain stem, were observed in 18 patients. Only two patients (number 3 and number 4), aged 8 and 10 years, had no abnormalities in WM. Hyperintensities were located predominantly in frontal periventricular white matter in 17 patients, followed by parietooccipital periventricular white matter in 9. Dense hyperintense lesions were observed in frontal region (11/17) as well as in parietooccipital region (7/9) (Figure 2). U-fibers of the frontal (7/20) and parietooccipital areas (6/20) were the second common locations of hyperintense lesions. Only one patient with maximal score (18) had periventricular hyperintensities, located in the temporal lobe.

**Figure 3:**
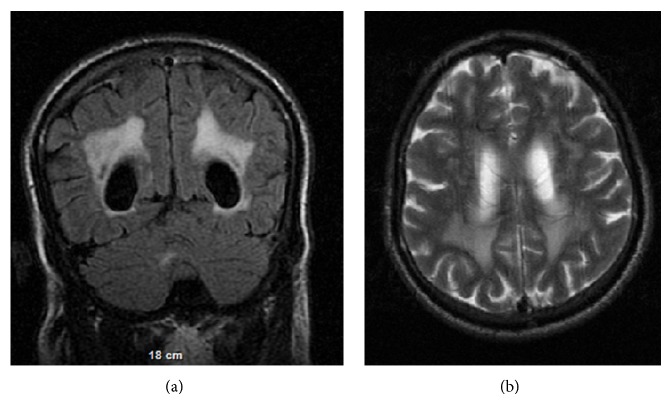
Patient number 19: a 46-year-old man with severity score 18. (a) Coronal FLAIR image shows bilateral dense parietal and occipital hyperintensities, confluent in the posterior periventricular areas and significant enlargement of ventricles. (b) Axial T2W image demonstrates round subcortical bilateral frontal white matter hyperintensities and significant enlargement of ventricles and subarachnoid spaces (severity of brain atrophy: 3 points).

The MRI severity score of the patients varied from 0 to 18. Three patients (number 16, number 18, and number 19), aged 37 to 46 years, had scores greater than 10. Only one patient (number 19) with the maximal MR imaging severity score (18) had hyperintensities in the midline pons. Neuroimaging in this 46-year-old patient showed bilateral dense frontal, parietal, and occipital hyperintensities confluent in the posterior periventricular areas, round subcortical bilateral frontal and parietal hyperintensities, and significant enlargement of ventricles and subarachnoid spaces (Figure 3).

The correlation analysis of the structural changes of the central nervous system and the cognitive impairment found a statistically significant correlation only between the impairment of short-term verbal memory and the MRI changes (Table 6).

## 4. Discussion and Conclusions

CCFDN is a complex disorder whose major manifestations involve the anterior segment of the eye, the skull and face, the peripheral nerves, and the endocrine system. Its pathogenesis involves basic cellular mechanisms of gene expression, as CTDP1 mutation is expressed in all cell types [7]. This disorder, known to impair RNA polymerase II-mediated gene expression, seems to affect the central nervous system (CNS) as well. The main differential diagnosis of CCFDN is Marinesco-Sjögren syndrome (MSS), characterized by the tetralogy of cerebellar ataxia, congenital cataracts, intellectual disability, and progressive muscle weakness due to myopathy.

In line with previous findings [1, 4, 6, 9] the cognitive impairment of our CCFDN cohort was limited to either borderline intelligence or mild mental retardation. None of the patients had normal intelligence or moderate to severe mental retardation. Neuropsychological involvement was present since childhood with delay in starting to talk in the majority of the affected patients. All the evaluated cognitive domains (general intelligence, verbal memory, executive functions, and language) seemed similarly affected. Bearing in mind that there was no significant correlation between cognitive impairment on one hand and age on the other, we can speculate that cognitive impairment is not progressive, as is the neuropathy. Of course longitudinal follow-up is needed to make such conclusions.

The cognitive impairment in CCFDN seems quite homogeneous, limited to borderline intelligence or mild mental retardation and language abilities, developed at a concrete and pragmatic level, whereas MSS patients have a large spectrum of neuropsychological involvement, ranging from normal intelligence to severe mental retardation [16, 17].

Other signs of CNS involvement previously reported by Müllner-Eidenböck et al. [8] in terms of, for example, the presence of pyramidal signs, extrapyramidal hyperkinesia, and ataxia were observed in 5 CCFDN patients from our group.

Although demyelinating peripheral neuropathy has been extensively studied in patients with CCFDN syndrome [1, 9], structural changes of the central nervous system are still to be elucidated [1, 6, 9, 18].

The main abnormalities on brain MRI include diffuse cerebral atrophy, enlargement of the lateral ventricles, and focal lesions in the subcortical white matter, different in number and size, consistent with demyelination more pronounced in the older CCFDN patients. MRI appearance of patients with CCFDN was not compatible with previously described imaging pattern of hypomyelinating disorders [19]. Cerebellar atrophy, considered as a MRI hallmark of MSS patients, is not typical for CCFDN, as it was observed in only one patient from our cohort, who also had global cerebral atrophy. Periventricular white matter changes present in both disorders are much rarer in MSS than in CCFDN [17, 20, 21].

In contrast to the hypomyelination in the peripheral nerves in our series of CCFDN patients no typical features of hypomyelination or delayed myelination were observed [22, 23]. All adult patients (older than 18 years) in our group have gained complete brain WM myelination. Five younger patients aged between 3 and 13 years had some terminal zones in the temporal and frontal subcortical regions which were not completely myelinated. These changes can be considered insignificant according to patients' age, because terminal zones do not stain for myelin until the fourth decade of life [24]. One of the study limitations related to hypomyelination is the broad age range of the study group, as well as the fact that the brain MRI except once was performed after the age of 8 years. Although we could not find evidence of hypomyelination on the conventional MRI, such cannot be excluded at an earlier age. We can suppose that in CCFDN a hypo/demyelination mechanism underlies cerebral WM damage similar to peripheral nervous system [9, 18]. We emphasize the importance of neuroimaging follow-up for better understanding of the precise nature and evolution of brain lesions.

In this study we applied a severity score system for brain MRI findings in an attempt to find a correlation with cognitive impairment. This scoring system could be of use in future neuroimaging studies, as well as in assessing lesion progression.

## Figures and Tables

**Table 1:** Brain imaging scoring system [11].

Brain areas	Score				Maximum per area
Frontal WM					6
Periventricular	0	1	2		
Central	0	1	2		
U-fibers	0	1	2		
Parietooccipital WM					6
Periventricular	0	1	2		
Central	0	1	2		
U-fibers	0	1	2		
Temporal WM					6
Periventricular	0	1	2		
Central	0	1	2		
U-fibers	0	1	2		
Corpus callosum					4
Genu	0	1	2		
Splenium	0	1	2		
Projection fibers					6
Internal capsule posterior limb	0	1	2		
Internal capsule anterior limb	0	1	2		
Midline pons	0	1	2		
Cerebral atrophy	0	1	2	3	3
Cerebellum					2
WM	0	1			
Atrophy	0	1			

Total					33

**Table 2:** Clinical features of CCFDN patients.

Patient *N*	Age at evaluation (years)	Age of starting to walk (years)	Age of starting to talk (years)	Functional Disability Scale (FDS)^∗^	Klockgether ataxia score^∗∗^
1	4	1,8	2,5	4	2
2	8	2,5	1,5	1	11
3	8	2	3	2	0
4	9	3	2	4	10
5	12	2	2	2	0
6	13	Independent ambulation, never achieved	3	7	0
7	13	4	4	2	5
8	16	2,5	1,3	5	0
9	16	2	2	3	8
10	17	2	2	2	1
11	18	4	4	3	5
12	19	5	4	7	7
13	24	5	12	3	2
14	26	1,2	2	3	3
15	30	2	2,5	3	4
16	33	1,5	2,8	3	4
17	34	2	2	4	5
18	37	3	3	3	8
19	42	2	2	3	5
20	45	2,5	2,5	2	0
21	46	6	3	4	5
22	47	2	2,5	3	1

^∗^Functional Disability Scale [12], ranging from 0 to 8 (0: normal, 8: bedridden).

^∗∗^Klockgether ataxia score [13], for measuring dysarthria, ataxia of walk, stance, upper and lower extremities, dysdiadochokinesis, and intention tremor on a scale from 0 to 5 for each item (0: best, 5: worst). The whole evaluation ranges from 0 to 35.

**Table 3:** Comparative analysis of the results of the neuropsychological tests between patients with CCFDN and healthy control group.

Test	Healthy controls			CCFDN patients			*p*
	*n*	$\overset{-}{X}$	SD	*n*	$\overset{-}{X}$	SD	
IQ	24	**78,13**	10,51	22	60,95	7,90	<0,001
RАVLT immediate recall, total	24	**44,58**	5,41	22	32,86	8,55	<0,001
RАVLT delayed recall	24	**9,38**	1,74	22	5,36	2,74	<0,001
RAVLT recognition	24	**28,38**	1,84	22	22,55	5,58	<0,001
TOL, total move score	24	**50,79**	21,73	22	58,27	12,91	n.s.
TOL, total correct score	24	**3,96**	1,40	22	1,77	1,31	<0,001
TOL, total rule violation	24	**0,63**	0,65	22	3,82	2,04	<0,001
TOL, total time violation	24	**0,92**	1,02	22	3,77	1,48	<0,001
TOL, initiation time	24	**69,13**	13,81	22	78,82	33,13	n.s.
TOL, execution time	24	**277,17**	57,50	22	482,77	111,33	<0,001
TOL, total problem solving time	24	**345,13**	63,02	22	565,50	116,97	<0,001
Semantic verbal fluency “animals”	24	**14,38**	4,24	22	8,36	3,42	<0,001
Phonemic verbal fluency “M”	24	**4,08**	1,79	22	1,91	1,80	<0,001

RAVLT: Rey Auditory Verbal Learning Test, TOL: Tower of London.

**Table 4:** Correlation coefficients between age, FDS, measuring severity of motor impairment, and the results from the neuropsychological tests in CCFDN patients.

	Age	FDS
RАVLT immediate recall	0,227	−0,226
RАVLT delayed recall	−0,008	−0,225
RАVLT recognition	0,272	−0,328
TOL, total move score	−0,025	0,164
TOL, total correct score	0,253	−0,147
TOL, total rule violation	−0,115	−0,035
TOL, total time violation	−0,396	0,323
TOL, initiation time	0,016	0,365
TOL, execution time	0,141	−0,034
TOL, total problem solving time	0,206	0,027
Semantic verbal fluency “animals”	0,407	−0,314
Phonemic verbal fluency “M”	0,199	−0,501

RAVLT: Rey Auditory Verbal Learning Test, TOL: Tower of London.

**Table 5:** Summary of MR imaging features in the CCFDN group.

Patient *N*	Age at MRI	Periventricular WM changes	Projection fibers' involvement	Cerebral atrophy	Cerebellar atrophy	MRI severity score
1	3	Yes	No	No	No	2
2	8	Yes	No	Yes	No	5
3	8	No	No	No	No	0
4	9	No	No	Yes	No	1
5	12	Yes	No	Yes	No	3
6	13	Yes	No	Yes	No	6
7	13	Yes	No	Yes	No	7
8	16	Yes	No	Yes	No	6
9	17	Yes	Yes	Yes	No	7
10	18	Yes	No	Yes	No	5
11	24	Yes	No	Yes	No	8
12	26	Yes	No	Yes	Yes	6
13	30	Yes	No	Yes	No	2
14	33	Yes	No	Yes	No	4
15	34	Yes	No	Yes	No	6
16	37	Yes	No	Yes	No	11
17	45	Yes	No	Yes	No	6
18	46	Yes	No	Yes	No	11
19	46	Yes	Yes	Yes	No	18
20	47	Yes	No	Yes	No	3

**Table 6:** Correlation coefficients between MRI severity score and the results of neuropsychological tests in patients with CCFDN.

	MRI severity score
**RAVLT immediate recall**	-0,405^∗^
RAVLT delayed recall	−0,288
RAVLT recognition	−0,408
TOL, total move score	0,180
TOL, total correct score	−0,153
TOL, total rule violation	−0,139
TOL, total time violation	−0,287
TOL, initiation time	−0,210
TOL, execution time	−0,230
TOL, total problem solving time	−0,152
Semantic verbal fluency “animals”	0,135
Phonemic verbal fluency “M”	−0,098

^∗^ *p* value < 0.05—statistically significant.
